# Learning needs analysis to guide teaching evidence-based medicine: knowledge and beliefs amongst trainees from various specialities

**DOI:** 10.1186/1472-6920-7-11

**Published:** 2007-05-10

**Authors:** Julie A Hadley, David Wall, Khalid S Khan

**Affiliations:** 1 Birmingham Women's Health Care NHS Trust, Metchley Park Road, Edgbaston, Birmingham, B15 2TG, UK; 2 The West Midlands Deanery, Birmingham Research Park, 97 Vincent Drive, Edgbaston, Birmingham, B15 2SQ, UK; 3 The University of Birmingham, Metchley Park Road, Edgbaston, Birmingham, B15 2TT, UK

## Abstract

**Background:**

We undertook a needs assessment exercise using questionnaire survey of junior doctors' knowledge and beliefs concerning evidence-based medicine (EBM) and critical literature appraisal, as this is a core competence in postgraduate medical education.

**Methods:**

We surveyed 317 junior doctors in various specialities in the UK West Midlands Deanery. Using validated questionnaires we compared the needs of different trainee groups. Results overall were internally consistent (Cronbach's alpha 0.929).

**Results:**

Respondents' generally felt that they had poor training in EBM (Mean score 2.2, possible range 1 – 6) and that they needed more education (Mean score 5.3, possible range 1–6). Male trainees felt more confident at evaluating statistical tests than females (p = 0.002). Female trainees considered patient choice above the evidence more often than males (p = 0.038). Trainees from surgical speciality felt more confident at assessing research evidence (p = 0.009) whereas those from medical speciality felt more confident at evaluating statistical tests (p = 0.038) than other specialities. However, non-surgical specialities tended to believe that EBM had little impact on practice (p = 0.029). Respondents who had been qualified for 11 years or over felt overall more confident in their knowledge relating to EBM than those who had been qualified less than 10 years. In particular, they felt more confident at being able to assess study designs (p = < 0.001) and the general worth of research papers (p = < 0.001). Trainees with prior research experience were less likely to find original work confusing (p = 0.003) and felt more confident that they can assess research evidence (p = < 0.001) compared to those without previous research experience. Trainees without previous research experience felt that clinical judgement was more important than evidence (p = < 0.001).

**Conclusion:**

There is a perceived deficit in postgraduate doctors' EBM knowledge and critical appraisal skills. Learning needs vary according to gender, place of basic medical qualification, time since graduation, prior research experience and speciality. EBM training curricular development should take into account the findings of our needs assessment study.

## Background

High quality health care implies clinical practice that is consistent with the current best evidence[1]. Evidence-based Medicine (EBM) has thus become an impetus for incorporating critical appraisal of research evidence alongside routine clinical practice. Increasingly, acquisition of knowledge and skills for EBM is becoming a core competence to be acquired by all doctors. The UK Foundation Programme Committee [2] states that trainee doctors should be able to understand, critically appraise and evaluate the evidence base for medical practice and implement available evidence into clinical care.

To develop relevant teaching and learning opportunities for trainee doctors, it is essential that a needs assessment exercise is undertaken, particularly because EBM is not uniformly taught in undergraduate education. The findings of such an exercise can provide critical evidence for development and tailoring of EBM curricula improving the effectiveness of teaching. We undertook such an exercise using questionnaire survey of postgraduate doctors' knowledge, skills and beliefs in various specialties in the UK West Midlands Deanery. This allowed us the opportunity to compare and contrast different needs of groups according to gender, specialty, place of basic qualification, time since qualification and previous research experience.

## Methods

During 2004–05 we surveyed all 317 junior doctors who attended one-day voluntary EBM workshops[3] prior to commencement of teaching. Attendance to these workshops was not a mandatory part of the junior doctors' training. The study was planned prospectively using recommended methods for educational needs analyses [4] and questionnaire surveys[5]. Ethical approval for the study was not required. Participants were made aware of the purpose of the survey, the anonymous nature of the dataset generated and the option to not respond if they so wished. This information served as the basis for an informed consent from each respondent.

### Questionnaire development

We developed a questionnaire to measure clinician's basic knowledge and beliefs concerning the main principles of EBM including questions from previously published and validated questionnaires [3,6,7]. This included assessment of literature searching behaviour, self perceived knowledge of critical appraisal skills and beliefs. Closed questions with multiple choice answers were used along with those seeking responses on six-point Likert scales. For example, participants were asked how often they searched for evidence? Participants selected responses from a range of options, which included the statements 'more than once a week', 'every 1–2 weeks', 'every 3–4 weeks'. 'less than once a month' and 'never'. Participants were asked how confident did they think they were at assessing various aspects of a published paper or how confident they felt that they were able to assess adequacy of sample size, ascertain bias and evaluate statistical tests. Answers ranged from '1' not confident at all to '6' very confident. Items on beliefs about EBM included statements such as 'EBM is essential in my practice', 'clinical judgement is more important than EBM' and 'I feel that I need more training in EBM'. Participants scored their answers on a range from '1' indicating that they disagreed strongly with the statement to '6' suggesting that they agreed strongly with the statement.

### Questionnaire administration and analysis

The questionnaires were self-administered by the candidates on arrival to the teaching session. All data obtained were entered into a Microsoft Excel spreadsheet and exported for analyses using SPSS versions 12.0.1 and 13.0. We used standard approaches to statistical analysis of questionnaire data including frequencies and descriptive summaries for the categorical data, and means, ranges, standard deviations and 95% confidence intervals for the Likert data [8].

Our analysis for internal consistency of the Likert scale questionnaire items used Cronbach's alpha, both overall and using the alpha for item deleted function, to look for "rogue" questions – that is questions answered in a quite different and inconsistent way. Cronbach's alpha assesses the internal consistency of the questionnaire results, that is, do the items to be measured look at much the same thing? There does not appear to be a consistent opinion on the value of Cronbach's alpha for scale reliability. An alpha of 0.5 or above is considered by Bowling [9] as an indication of good internal consistency, whereas an alpha of 0.7 or above is considered satisfactory by Howitt and Cramer [10]. We used a figure of 0.7, and our data for questions on knowledge about EBM had an alpha of 0.929 – a very high figure.

For comparisons of Likert scale data by other variables (such as gender, place of basic qualification, years since obtaining qualification, and speciality) we used non- parametric tests, the Mann Whitney test for two categories and Kruskall Wallis test for three or more categories. These are non-parametric tests, which Jamieson [11] and Cohen, Manion and Morrison [12] consider the most appropriate for Likert scale data. These data are ordinal, does have a rank order, but the intervals between values cannot be considered equal. However, it is becoming common practice to use ANOVA for the analysis of such data [13]. In addition, Pell [14] considers the use of ANOVA acceptable practice. In this study we used both categories of statistical tests on our Likert scale data, and in the text of the results below we only report the p-values for non-parametric tests. In most instances the results were similar.

## Results

### Participants

**Figure 1:**
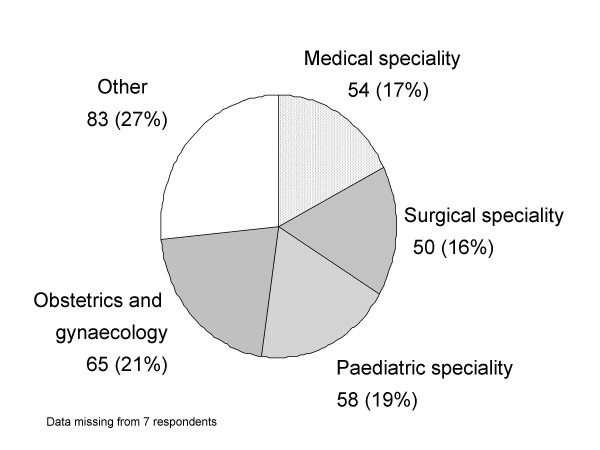
Breakdown of specialties of 317 trainee doctors participating in the Evidence-Based Medicine needs analysis questionnaire survey 2004–05.

All 317 clinicians approached completed the questionnaire (100% return). Of these, 181 (57%) were males and 135 (42.5%) were females. The respondents stated that the place where they obtained their basic medical qualification was the UK for 107 (33.8%), overseas (not including Europe) for 195 (61.5%) and Europe for 12 (3.8%). Furthermore, 162 (51.1%) had qualified within the last 10 years, whilst 136 (42.9%) had been qualified for 11 years and over. Data concerning distribution of specialties are shown in Figure 1.

The respondents' background of exposure to research and EBM showed that the majority of the respondents (246, 77.6%) had not attended a literature appraisal skills workshop. Only 24 (7.6%) of respondents stated that they had received formal training in research methods, six people (1.9%) had had epidemiology training and 24 (7.6%) had attended statistics courses. However, 192 (60.6%) stated that they had actually been personally involved in conducting some research activity.

Questions regarding clinicians' access to medical literature and evidence showed that all the clinicians except for two had access to a medical library and had access to medical literature on the Internet (n = 312). However, only 63 (19.9%) searched for medical literature more than once a week, 78 (24.6%) every 1–2 weeks, the rest searched less frequently or not at all. Furthermore, of all the respondents only 121 (38.2%) reported that they read every week regularly to keep up to date with their professional literature.

### Doctors' perceived knowledge and beliefs

**Figure 2:**
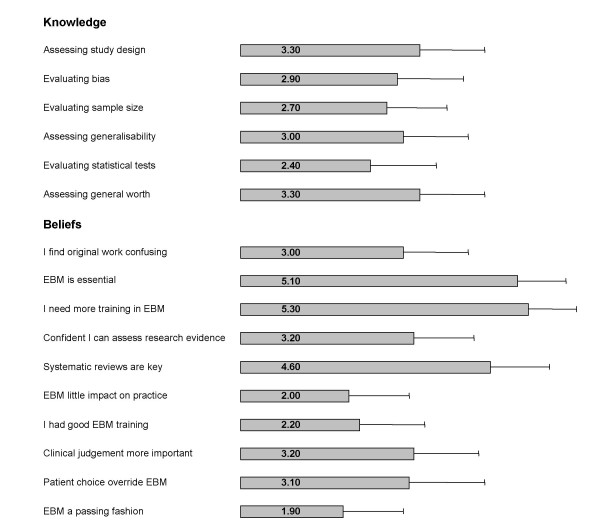
Trainee doctors' self-perceived knowledge and beliefs relating to Evidence-based Medicine (EBM). Responses measured on a 6-point Likert scale*. * Likert scale for self-perceived knowledge: '1' indicated no confidence at all while '6' indicating that respondents felt very confident. Likert scale for beliefs: '1' suggested that respondents disagreed with the statement while '6' indicating that they strongly agreed. Error bar indicates 95% confidence intervals. Data missing from some respondents for each item.

Figure 2 shows clinicians' self perceived knowledge and beliefs relating to EBM. Respondents' scores indicated that they did not feel confident at assessing study design, generalisability and general worth of a paper, or evaluating bias, sample size and statistical tests. These results overall were very consistent (Cronbach's alpha of 0.929 – a very high value). The majority of clinicians specified that they felt that they had not had good or adequate training in EBM (Mean score 2.2) and they identified that they needed more training and education in the principles of EBM (Mean score 5.3). Although, some confusion regarding the relationship between EBM and the process of clinical decision-making was found, with many clinicians feeling unsure whether their own clinical judgement and patient choice should override the evidence (Mean scores 3.2 and 3.1 respectively). However, the clinicians agreed that they felt that EBM was essential to their practice (Mean score 5) and not a passing fashion (Mean score 1.9).

### Doctors' gender, place of basic medical qualification and speciality

**Figure 3:**
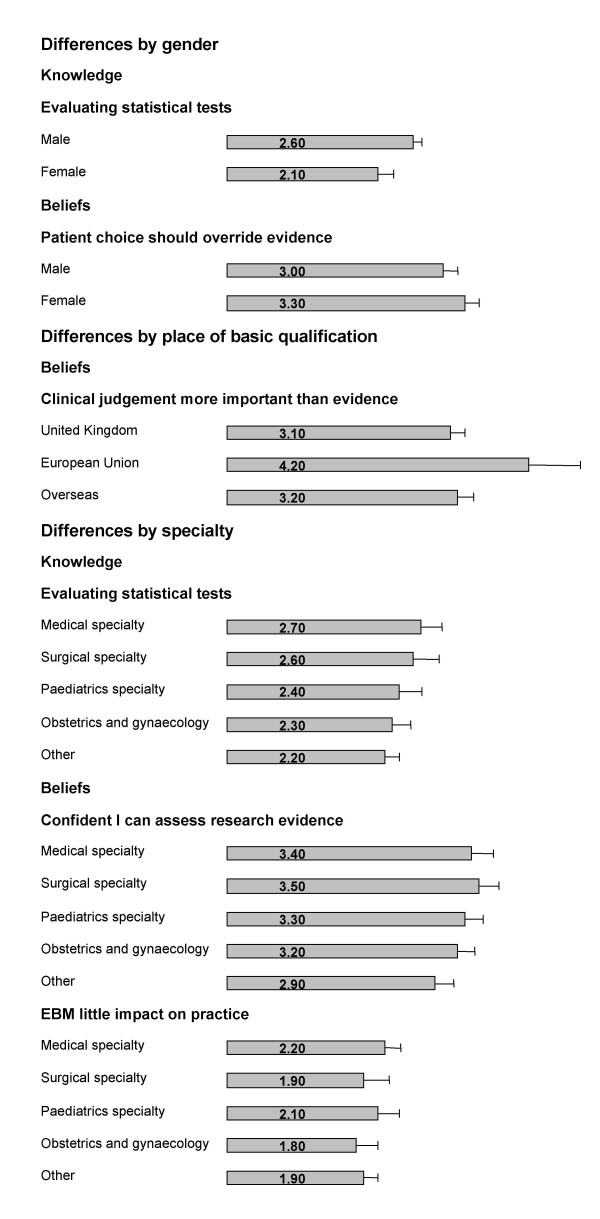
Effect of gender, place of basic medical qualification and specialty on trainee doctors' self perceived knowledge and beliefs relating to Evidence-based Medicine (EBM). Likert scale for self-perceived knowledge: '1' indicated no confidence at all while '6' indicating that respondents felt very confident. Likert scale for beliefs: '1' suggested that respondents disagreed with the statement while '6' indicating that they strongly agreed. * Only significant differences reported. Error bar indicates 95% confidence intervals. Data missing from some respondents for each item.

Figure 3 shows the effect of gender, place of basic medical qualification and speciality on the clinicians' self perceived knowledge and beliefs relating to EBM. It was found on subgroup analysis that male doctors felt more confident at evaluating statistical tests than female doctors (p = 0.002). But female doctors considered that patient choice should override the evidence more often than males (p = 0.038). Also clinicians who qualified in the UK were more likely to believe that clinical judgment is more important than the evidence (p = 0.009). Furthermore, trainees from surgical specialities felt more confident at assessing research evidence (p = 0.009), and medical speciality trainees felt more confident at evaluating statistical tests (p = 0.038) than those clinicians from other specialities. However, non-surgical specialities tended to believe that EBM had little impact on practice (p = 0.029).

### Effect of length of time since qualification

**Figure 4:**
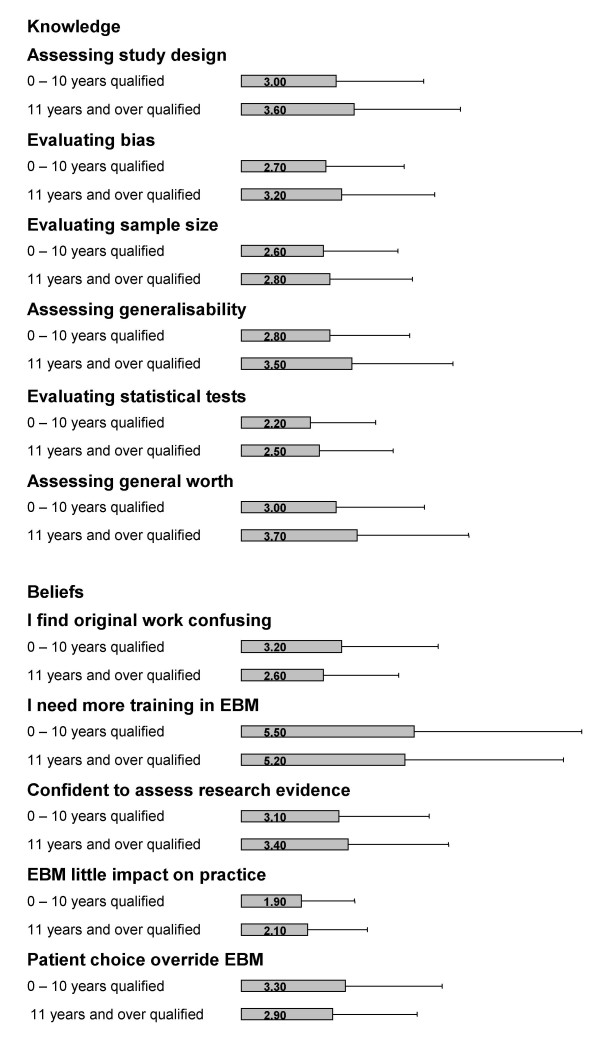
Effect of years since basic medical qualification (10 years and under vs 11 years and over) on trainee doctors' self perceived knowledge and beliefs relating to Evidence-based Medicine (EBM). Likert scale for self-perceived knowledge: '1' indicated no confidence at all while '6' indicating that respondents felt very confident. Likert scale for beliefs: '1' suggested that respondents disagreed with the statement while '6' indicating that they strongly agreed. *Only significant differences reported. Error bar indicates 95% confidence intervals. Data missing from some respondents for each item.

As shown in Figure 4, significant differences were also found when comparisons were made between the length of time since basic medical qualification was obtained. The respondents who had been qualified for 11 years or over felt overall more confident in their knowledge relating to EBM than those doctors who had been qualified less than 10 years. In particular, they felt more confident at being able to assess study designs (p = < 0.001) and assess the general worth of research papers (p = < 0.001). Furthermore, they felt that they were less likely to find original work confusing (p = < 0.001), but still identified that they needed more training in the principles of EBM.

### Effect of previous research experience

**Figure 5:**
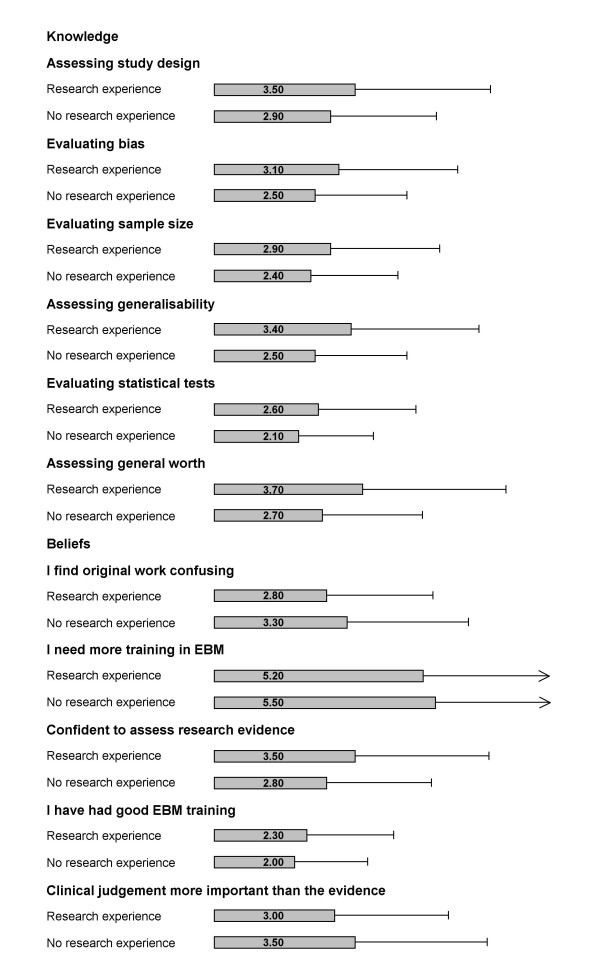
Effect of involvement in previous research on trainee doctors' self perceived knowledge and beliefs relating to Evidence-based Medicine (EBM). Likert scale for self-perceived knowledge: '1' indicated no confidence at all while '6' indicating that respondents felt very confident. Likert scale for beliefs: '1' suggested that respondents disagreed with the statement while '6' indicating that they strongly agreed. *Only significant differences reported. Error bar indicates 95% confidence intervals. Data missing from some respondents for each item.

Figure 5 examines the effect of involvement in previous research experience on trainee doctors' knowledge and beliefs relating to EBM. Those trainees with previous research experience scored considerably higher in all the questions relating to knowledge than those without. In particular, all results were highly statistical significant with p-values = < 0.001. Those with research experience were also less likely to find original work confusing (p = 0.003) and feel more confident that they can assess research evidence (p = < 0.001). Alternatively, those trainees with no previous research experience feel that clinical judgement is more important than the evidence (p = < 0.001).

For a more in-depth breakdown of the results please refer to the accompanying additional file 1.

## Conclusion

Our study identified several major issues that require addressing, not only in postgraduate medical education but also in the undergraduate curriculum. Many factors were found to be important determinants of improved EBM knowledge and appropriate attitudes. Learning needs varied according to gender, place of basic medical qualification, time since graduation, prior research experience and speciality.

Many key lessons have emerged for EBM teachers to consider carefully from our study. Many junior doctors support the principles of EBM, but they are undecided regarding whether patient choice and their own clinical judgment are more important and should override research evidence. Interestingly, those who had qualified overseas felt that clinical judgement was more important than the evidence compared to those who had qualified in the UK. Women and trainees without research experience gave greater emphasis to patient choice and clinical judgment, whereas men and those with previous research exposure value EBM and research more. Surgical trainees, those with previous research experience and those clinicians who had been qualified for over 11 years were more confident in assessing research studies. Trainees in medical specialities, those without research experience and those who had been qualified 10 years or less were less confident. We found that gender, speciality, time since qualification and previous research experience were important determinants of knowledge and beliefs. Elicitation of these variations is critical to planning curricula content and emphasis as prior knowledge and beliefs are key factors in determining learning achievement.

The validity and generalisability of our findings depends on the rigour of our questionnaire design, execution and analyses. We selected questions that had previously been validated and missing responses were few. One of the strengths of our study is that we surveyed a large sample of doctors from a variety of specialities and with varying lengths of time since qualification. However, our sampling strategy may be criticised, as it does not employ a random process. There are currently approximately 1000 postgraduate trainees in the West Midlands Deanery. We therefore, surveyed approximately 32% off all eligible trainees. Our respondents may have been more aware and self-motivated than trainees generally. If this is so, our findings provide a more conservative view of the training needs. We may also be criticised in methodological terms as the questionnaire relied on the doctors' self-perceived assessment of their own knowledge and beliefs [3,15]. Research participants may feel pressurised into completing the questionnaire, or unwilling to divulge knowledge and skill deficiencies [16]. Questionnaire research is never completely objective and as such potential biases can be introduced. We feel that our findings merit consideration by EBM educationalists, particularly those involved in postgraduate education.

Our survey substantiates and extends findings of previous studies by Olatunbosun et al [17] and Awonuga et al [3], who also found that clinicians lack methodological competence in critical appraisal skills and EBM. It concurs with previous findings that there is a deficit in postgraduate doctors' knowledge of EBM and critical appraisal skills, which requires addressing through education as the majority of trainees feel unconfident in their ability to assess research studies. Additionally, it provides information to tailor teaching curricula to the needs of specific subgroups of trainees. The overall aims and objectives of existing teaching courses need not change, but teaching methods need to be adapted to student's profiles that suggest a particular learning need. Thus, when planning teaching careful attention should be paid to the factors identified in this study.

## Competing interests

The authors declare no competing interests. The authors have received funding to teach and undertake research in evidence-based medicine.

## Authors' contributions

JH contributed to the questionnaire design, co-ordination and collection of the data and drafted the manuscript. DW participated in the questionnaire design, performed the statistical analysis and contributed to the write up of the manuscript. KK conceived the study, and participated in its design and helped to draft the manuscript. All authors read and approved the final manuscript.

## Pre-publication history

The pre-publication history for this paper can be accessed here:



## Supplementary Material

Additional file 1 Table 1. Trainee doctors' self-perceived knowledge and beliefs relating to Evidence-based Medicine (EBM). Responses measured on a 6-point Likert scale*.
Table 2. Effect of gender, place of basic medical qualification and specialty on trainee doctors' self perceived knowledge and beliefs relating to Evidence-based Medicine (EBM).
Table 3: Effect of years since basic medical qualification (10 years and under vs 11 years and over) on trainee doctors' self perceived knowledge and beliefs relating to Evidence-based Medicine (EBM).
Table 4: Effect of involvement in previous research on trainee doctors' self perceived knowledge and beliefs relating to Evidence-based Medicine (EBM). Click here for file
