# Supplementary material for: Learning needs analysis to guide teaching evidence-based medicine: knowledge and beliefs amongst trainees from various specialities
Source: BMC Med Educ. 2007 May 10;7:11. doi: 10.1186/1472-6920-7-11 (PMC1885246; doi:10.1186/1472-6920-7-11)
Supplement: Additional file 1 — Table 1. Trainee doctors' self-perceived knowledge and beliefs relating to Evidence-based Medicine (EBM). Responses measured on a 6-point Likert scale*. Table 2. Effect of gender, place of basic medical qualification and specialty on trainee doctors' self perceived knowledge and beliefs relating to Evidence-based Medicine (EBM). Table 3: Effect of years since basic medical qualification (10 years and under vs 11 years and over) on trainee doctors' self perceived knowledge and beliefs relating to Evidence-based Medicine (EBM). Table 4: Effect of involvement in previous research on trainee doctors' self perceived knowledge and beliefs relating to Evidence-based Medicine (EBM). [file 1472-6920-7-11-S1.doc]

**Table 1. Trainee doctors’ self-perceived knowledge and beliefs relating to Evidence-based Medicine (EBM). Responses measured on a 6-point Likert scale*.**

| **Question** | **N** | **Mean** | **Std. Deviation** |
| --- | --- | --- | --- |
| **Knowledge** |  |  |  |
| Assessing study design | 316 | 3.3 | 1.2 |
| Evaluating bias | 315 | 2.9 | 1.2 |
| Evaluating sample size | 314 | 2.7 | 1.1 |
| Assessing generalisability | 288 | 3.0 | 1.2 |
| Evaluating statistical tests | 315 | 2.4 | 1.2 |
| Assessing general worth | 314 | 3.3 | 1.2 |
| **Beliefs** |  |  |  |
| I find original work confusing | 284 | 3.0 | 1.2 |
| EBM is essential | 314 | 5.1 | 0.9 |
| I need more training in EBM | 315 | 5.3 | 0.9 |
| Confident I can assess research evidence | 316 | 3.2 | 1.1 |
| Systematic reviews are key | 307 | 4.6 | 1.1 |
| EBM little impact on practice | 317 | 2.0 | 1.1 |
| I had good EBM training | 312 | 2.2 | 1.2 |
| Clinical judgement more important | 313 | 3.2 | 1.2 |
| Patient choice override EBM | 311 | 3.1 | 1.4 |
| EBM a passing fashion | 307 | 1.9 | 1.1 |

Data missing from some respondents for each item

* Likert scale for self-perceived knowledge: ‘1’ indicated no confidence at all while ‘6’ indicating that respondents felt very confident. Likert scale for beliefs: ‘1’ suggested that respondents disagreed with the statement while ‘6’ indicating that they strongly agreed.

**Table 2. Effect of gender, place of basic medical qualification and specialty on trainee doctors’ self perceived knowledge and beliefs relating to Evidence-based Medicine (EBM).**

| **Subgroup and Question*** | **N** | **Mean** | **95% confidence intervals** | **P value ANOVA** | **P value**  **Mann Whitney or Kruskall Wallis** |
| --- | --- | --- | --- | --- | --- |
|  |  |  |  |  |  |
| **Differences by gender** |  |  |  |  |  |
| **Knowledge** |  |  |  |  |  |
| Evaluating statistical tests |  |  |  |  |  |
| Male | 180 | 2.6 | 2.38 – 2.72 | 0.044 | 0.002 |
| Female | 134 | 2.1 | 1.95 – 2.32 |  |  |
|  |  |  |  |  |  |
| **Beliefs** |  |  |  |  |  |
| Patient choice should override EBM |  |  |  |  |  |
| Male | 177 | 3.0 | 2.78 – 3.20 | 0.063 | 0.038 |
| Female | 133 | 3.3 | 3.06 – 3.51 |  |  |
|  |  |  |  |  |  |
| **Differences by place of basic qualification** |  |  |  |  |  |
| **Beliefs** |  |  |  |  |  |
| Clinical judgement more important than EBM |  |  |  |  |  |
| United Kingdom | 107 | 3.1 | 2.87 – 3.30 | 0.016 | 0.009 |
| European Union | 12 | 4.2 | 3.41 – 4.92 |  |  |
| Overseas | 191 | 3.2 | 3.05 – 3.42 |  |  |
|  |  |  |  |  |  |
| **Differences by specialty** |  |  |  |  |  |
| **Knowledge** |  |  |  |  |  |
| Evaluating statistical tests |  |  |  |  |  |
| Medical specialty | 54 | 2.7 | 2.24 – 2.99 | 0.050 | 0.038 |
| Surgical specialty | 50 | 2.6 | 2.33 – 2.95 |  |  |
| Paediatrics specialty | 57 | 2.4 | 2.09 – 2.72 |  |  |
| Obstetrics and gynaecology | 65 | 2.3 | 1.97 – 2.55 |  |  |
| Other | 82 | 2.2 | 1.92 – 2.40 |  |  |
|  |  |  |  |  |  |
| **Beliefs** |  |  |  |  |  |
| Confident I can assess research evidence |  |  |  |  |  |
| Medical specialty | 53 | 3.4 | 3.07 – 3.70 | 0.012 | 0.009 |
| Surgical specialty | 50 | 3.5 | 3.30 – 3.78 |  |  |
| Paediatrics specialty | 58 | 3.3 | 2.99 – 3.56 |  |  |
| Obstetrics and gynaecology | 65 | 3.2 | 2.90 – 3.44 |  |  |
| Other | 83 | 2.9 | 2.68 – 3.15 |  |  |
|  |  |  |  |  |  |
| EBM little impact on practice |  |  |  |  |  |
| Medical specialty | 54 | 2.2 | 1.91 – 2.42 | 0.436 | 0.029 |
| Surgical specialty | 50 | 1.9 | 1.66 – 2.26 |  |  |
| Paediatrics specialty | 58 | 2.1 | 1.71 – 2.39 |  |  |
| Obstetrics and gynaecology | 65 | 1.8 | 1.50 – 2.10 |  |  |
| Other | 83 | 1.9 | 1.72 – 2.09 |  |  |
|  |  |  |  |  |  |

Data missing from some respondents for each item

Likert scale for self-perceived knowledge: ‘1’ indicated no confidence at all while ‘6’ indicating that respondents felt very confident. Likert scale for beliefs: ‘1’ suggested that respondents disagreed with the statement while ‘6’ indicating that they strongly agreed.

* Only significant differences reported

**Table 3: Effect of years since basic medical qualification (10 years and under vs 11 years and over)** **on trainee doctors’ self perceived knowledge and beliefs relating to Evidence-based Medicine (EBM).**

| **Question*** | **n** | **Mean** | **95% confidence intervals** | **P value ANOVA** | **P value**  **Mann Whitney or Kruskall Wallis** |
| --- | --- | --- | --- | --- | --- |
|  |  |  |  |  |  |
| **Knowledge** |  |  |  |  |  |
| Assessing study design |  |  |  |  |  |
| 0 – 10 years qualified | 162 | 3.0 | 2.80 – 3.17 | <0.001 | <0.001 |
| 11 years and over qualified | 136 | 3.6 | 3.37 – 3.77 |  |  |
|  |  |  |  |  |  |
| Evaluating bias |  |  |  |  |  |
| 0 – 10 years qualified | 162 | 2.7 | 2.49 – 2.85 | <0.001 | 0.001 |
| 11 years and over qualified | 135 | 3.2 | 2.96 – 3.35 |  |  |
|  |  |  |  |  |  |
| Evaluating sample size |  |  |  |  |  |
| 0 – 10 years qualified | 161 | 2.6 | 2.38 – 2.74 | 0.040 | 0.036 |
| 11 years and over qualified | 136 | 2.8 | 2.64 – 3.01 |  |  |
|  |  |  |  |  |  |
| Assessing generalisability |  |  |  |  |  |
| 0 – 10 years qualified | 149 | 2.8 | 2.57 – 2.93 | <0.001 | <0.001 |
| 11 years and over qualified | 122 | 3.5 | 3.23 – 3.68 |  |  |
|  |  |  |  |  |  |
| Evaluating statistical tests |  |  |  |  |  |
| 0 – 10 years qualified | 161 | 2.2 | 2.06 – 2.42 | 0.031 | 0.022 |
| 11 years and over qualified | 136 | 2.5 | 2.34 – 2.72 |  |  |
|  |  |  |  |  |  |
| Assessing general worth |  |  |  |  |  |
| 0 – 10 years qualified | 160 | 3.0 | 2.82 – 3.18 | < 0.001 | < 0.001 |
| 11 years and over qualified | 136 | 3.7 | 3.54 – 3.91 |  |  |
|  |  |  |  |  |  |
| **Beliefs** |  |  |  |  |  |
| I find original work confusing |  |  |  |  |  |
| 0 – 10 years qualified | 143 | 3.2 | 3.05 – 3.43 | < 0.001 | < 0.001 |
| 11 years and over qualified | 125 | 2.6 | 2.41 – 2.84 |  |  |
|  |  |  |  |  |  |
| I need more training in EBM |  |  |  |  |  |
| 0 – 10 years qualified | 161 | 5.5 | 5.32 – 5.57 | 0.010 | 0.006 |
| 11 years and over qualified | 135 | 5.2 | 5.03 – 5.34 |  |  |
|  |  |  |  |  |  |
| Confident to assess research evidence |  |  |  |  |  |
| 0 – 10 years qualified | 162 | 3.1 | 2.88 – 3.20 | 0.005 | 0.007 |
| 11 years and over qualified | 136 | 3.4 | 3.20 – 3.56 |  |  |
|  |  |  |  |  |  |
| EBM little impact on practice |  |  |  |  |  |
| 0 – 10 years qualified | 162 | 1.9 | 1.71 – 2.05 | 0.117 | 0.035 |
| 11 years and over qualified | 136 | 2.1 | 1.90 – 2.27 |  |  |
|  |  |  |  |  |  |
| Patient choice override EBM |  |  |  |  |  |
| 0 – 10 years qualified | 159 | 3.3 | 3.08 – 3.49 | 0.026 | 0.014 |
| 11 years and over qualified | 133 | 2.9 | 2.68 – 3.17 |  |  |
|  |  |  |  |  |  |

Data missing from some respondents for each item

Likert scale for self-perceived knowledge: ‘1’ indicated no confidence at all while ‘6’ indicating that respondents felt very confident. Likert scale for beliefs: ‘1’ suggested that respondents disagreed with the statement while ‘6’ indicating that they strongly agreed.

*Only significant differences reported.

**Table 4: Effect of involvement in previous research on trainee doctors’ self perceived knowledge and beliefs relating to Evidence-based Medicine (EBM).**

| **Question*** | **n** | **Mean** | **95% confidence intervals** | **P value ANOVA** | **P value**  **Mann Whitney or Kruskall Wallis** |
| --- | --- | --- | --- | --- | --- |
|  |  |  |  |  |  |
| **Knowledge** |  |  |  |  |  |
| Assessing study design |  |  |  |  |  |
| Research experience | 192 | 3.5 | 3.37-3.69 | <0.001 | **<0.001** |
| No research experience | 121 | 2.9 | 2.64-3.08 |  |  |
|  |  |  |  |  |  |
| Evaluating bias |  |  |  |  |  |
| Research experience | 191 | 3.1 | 2.96-3.28 | <0.001 | **<0.001** |
| No research experience | 121 | 2.5 | 2.30-2.69 |  |  |
|  |  |  |  |  |  |
| Evaluating sample size |  |  |  |  |  |
| Research experience | 192 | 2.9 | 2.71-3.03 | <0.001 | **<0.001** |
| No research experience | 119 | 2.4 | 2.17-2.57 |  |  |
|  |  |  |  |  |  |
| Assessing generalisability |  |  |  |  |  |
| Research experience | 178 | 3.4 | 3.20-3.55 | <0.001 | **<0.001** |
| No research experience | 109 | 2.5 | 2.29-2.70 |  |  |
|  |  |  |  |  |  |
| Evaluating statistical tests |  |  |  |  |  |
| Research experience | 191 | 2.6 | 2.42-2.76 | <0.001 | **<0.001** |
| No research experience | 121 | 2.1 | 1.86-2.24 |  |  |
|  |  |  |  |  |  |
| Assessing general worth |  |  |  |  |  |
| Research experience | 192 | 3.7 | 3.57-3.86 | <0.001 | **<0.001** |
| No research experience | 119 | 2.7 | 2.49-2.92 |  |  |
|  |  |  |  |  |  |
| **Beliefs** |  |  |  |  |  |
| I find original work confusing |  |  |  |  |  |
| Research experience | 177 | 2.8 | 2.64-3.00 | 0.003 | **0.003** |
| No research experience | 104 | 3.3 | 3.03-3.50 |  |  |
|  |  |  |  |  |  |
| I need more training in EBM |  |  |  |  |  |
| Research experience | 191 | 5.2 | 5.06-5.33 | 0.011 | **0.002** |
| No research experience | 121 | 5.5 | 5.31-5.63 |  |  |
|  |  |  |  |  |  |
| Confident to assess research evidence |  |  |  |  |  |
| Research experience | 192 | 3.5 | 3.33-3.61 | <0.001 | **<0.001** |
| No research experience | 121 | 2.8 | 2.60-2.97 |  |  |
|  |  |  |  |  |  |
| I have had good EBM training |  |  |  |  |  |
| Research experience | 189 | 2.3 | 2.17-2.52 | 0.013 | **0.012** |
| No research experience | 120 | 2.0 | 1.81-2.19 |  |  |
|  |  |  |  |  |  |
| Clinical judgement more important than EBM |  |  |  |  |  |
| Research experience | 188 | 3.0 | 2.84-3.18 | <0.001 | **<0.001** |
| No research experience | 122 | 3.5 | 3.30-3.77 |  |  |
|  |  |  |  |  |  |

Data missing from some respondents for each item

Likert scale for self-perceived knowledge: ‘1’ indicated no confidence at all while ‘6’ indicating that respondents felt very confident. Likert scale for beliefs: ‘1’ suggested that respondents disagreed with the statement while ‘6’ indicating that they strongly agreed.

*Only significant differences reported.
